# A Methodology for Evaluating User Experience in Human-Centered Extended Reality Applications

**DOI:** 10.3390/biomimetics11030182

**Published:** 2026-03-03

**Authors:** Daniela Quiñones, Luis Felipe Rojas, Renato Olavarría, Claudio Cubillos, Felipe Muñoz-La Rivera

**Affiliations:** 1 Escuela de Ingeniería Informática, Pontificia Universidad Católica de Valparaíso, Valparaíso 2340000, Chile; flavio.olavarria.l@mail.pucv.cl (R.O.); claudio.cubillos@pucv.cl (C.C.); 2 Departamento de Electrotecnia e Informática, Universidad Técnica Federico Santa María, Viña del Mar 2520000, Chile; luis.rojasco@usm.cl; 3 Escuela de Ingeniería Civil, Pontificia Universidad Católica de Valparaíso, Valparaíso 2340000, Chile; felipe.munoz@pucv.cl

**Keywords:** user experience, extended reality, virtual reality, augmented reality, methodology, human-centered design, user experience evaluation, evaluation instruments

## Abstract

Extended Reality (XR) technologies are increasingly used to create immersive and interactive systems across domains such as education, training, health, and entertainment. As these systems become more complex and multisensory, evaluating user experience (UX) in XR environments requires approaches that go beyond traditional usability assessments and consider perceptual, cognitive, emotional, and interaction-related factors. However, existing UX evaluation efforts in XR often rely on isolated instruments or domain-specific studies, lacking a systematic and reusable evaluation methodology. This paper proposes a human-centered methodology for evaluating user experience in extended reality applications, integrating UX dimensions and XR-specific characteristics into a structured and coherent evaluation process. The methodology is grounded in a multi-phase research process that includes a comprehensive literature review, expert consultation, correlation analysis between UX dimensions and XR features, and formal specification of evaluation phases and activities. Based on this process, the proposed methodology supports evaluators in selecting appropriate UX evaluation methods and instruments according to the characteristics and experiential goals of XR applications. The methodology defines a set of UX dimensions tailored to immersive environments, capturing perceptual, cognitive, emotional, and interaction aspects that are critical for the design and evaluation of adaptive and human-centered XR systems. An expert-based validation was conducted to assess the clarity, usefulness, and applicability of the methodology, leading to refinements in its structure and descriptions. The methodology promotes a human-centered approach by considering user perception, emotional impact, and contextual experience across XR modalities. It additionally contributes to the field by offering a reusable process for UX evaluation in XR, supporting more consistent, transparent, and human-centered assessment practices. It also provides a foundation for future empirical studies and the development of evaluation approaches inspired by natural and adaptive human–environment interactions.

## 1. Introduction

Extended Reality (XR), encompassing Virtual Reality (VR), Augmented Reality (AR), and Mixed Reality (MR), has become a key technology for the design of immersive and interactive systems across domains such as education, training, industry, healthcare, and entertainment. Recent advances in XR hardware and software (including head-mounted displays and spatial computing devices) have enabled increasingly sophisticated multisensory experiences, transforming the ways users perceive, interact with, and engage with digital environments.

As XR systems evolve toward higher levels of immersion and interactivity, the evaluation of user experience (UX) becomes essential to ensuring that these applications are not only functional, but also usable, engaging, emotionally meaningful, and cognitively sustainable. UX in XR environments involves complex perceptual, cognitive, emotional, and interaction-related processes, many of which extend beyond the scope of traditional desktop or mobile interfaces. However, many widely used UX evaluation methods and instruments were originally developed for conventional interaction paradigms and may be insufficient to capture the experiential complexity inherent to immersive environments. To address these limitations, existing studies in XR often combine multiple UX evaluation instruments within a single evaluation effort to assess usability, presence, workload, immersion, or emotional response [1]. While this strategy provides richer insights, it frequently lacks a systematic rationale for instrument selection and integration, leading to fragmented evaluation practices and limited comparability across studies. This situation highlights the need for structured UX evaluation methodologies that explicitly consider the distinctive features of XR applications, such as immersion, embodiment, multisensory interaction, environmental feedback, and adaptive system behavior.

From a biomimetic perspective, XR systems constitute a particularly relevant context for human-centered interaction research. Like biological systems, immersive technologies rely on the integration of multisensory information, continuous feedback loops, and real-time adaptation to dynamic environments. Human perception and cognition (shaped by evolutionary processes) provide valuable inspiration for designing and evaluating interactive systems that align with natural perceptual and behavioral capabilities. In this sense, UX evaluation in XR can benefit from approaches that consider interaction as an adaptive, embodied, and context-sensitive process, rather than a purely task-oriented activity.

Despite the growing interest in UX evaluation for XR, there is still a lack of comprehensive and reusable methodologies that integrate UX dimensions, XR-specific characteristics, and evaluation instruments into a coherent and decision-oriented process. Existing approaches often focus on isolated aspects of the experience or on specific application domains, limiting their generalizability and practical adoption.

To address this gap, this article proposes a methodology for evaluating user experience in human-centered extended reality applications. The proposed methodology integrates multiple UX dimensions and XR characteristics into a structured evaluation process and provides guidance for selecting appropriate evaluation methods and instruments according to the nature and goals of XR applications. The methodology was developed through a rigorous eight-phase research process, including an extensive literature review, expert consultation, correlation analysis, formal specification, and expert-based validation. The main contributions of this work are: (1) the proposal of a structured and reusable UX evaluation methodology for XR applications; (2) the definition of UX dimensions tailored to immersive and adaptive XR environments; (3) the introduction of a decision-oriented methodology to support the selection of UX evaluation methods and instruments in XR; and (4) an expert-based validation demonstrating the clarity, usefulness, and applicability of the proposed methodology.

The remainder of this paper is organized as follows. Section 2 reviews the background and related work on UX evaluation in XR and human-centered interaction. Section 3 describes the process for developing the methodology. Section 4 details the results obtained throughout each phase of the methodological development process (including its validation). Section 5 presents the UX evaluation methodology and its phases. Section 6 discusses the implications of the methodology from a human-centered and biomimetic perspective, as well as its limitations. Finally, Section 7 concludes the paper and includes the future research directions.

## 2. Background and Related Work

UX evaluation in XR environments has gained increasing attention in recent years. It is no longer sufficient to assess isolated aspects such as usability, interaction, or satisfaction; instead, more holistic evaluations are required; ones that account for cognitive, emotional, sensory, and contextual dimensions. Accordingly, UX in XR is often evaluated through a combination of questionnaires, interviews, direct observation, and user testing.

### 2.1. User Experience (UX)

Specifically, UX refers to the perceptions, emotions, and responses that arise from the use (or anticipated use) of a product, system, or service. It is influenced by factors such as usability, efficiency, and emotional impact. Unlike usability (which focuses on task completion and effectiveness) UX encompasses a broader and more subjective evaluation, including aesthetic, hedonic, and affective dimensions [2]. From a biomimetic standpoint, UX can be enriched by observing how biological systems interact with their environments efficiently, adaptively, and emotionally. Understanding UX as a multidimensional construct aligns with nature-inspired design, where the user’s functional, perceptual, and emotional responses mirror the complex ways living organisms respond to stimuli.

Various models have been developed to conceptualize UX. For example, Hassenzahl [3] distinguishes between pragmatic qualities (e.g., functionality and efficiency) and hedonic qualities (e.g., stimulation, identification, and aesthetics). In contrast, Morville [4] proposes seven key factors that influence UX: useful, usable, desirable, findable, accessible, credible, and valuable. In addition, Jean-Marc Robert [5] defines UX across the following eight dimensions: Functional, Usefulness/Usability, Informational, Physical characteristics, Cognitive, Psychological, and Social. These dimensions are particularly relevant in immersive systems (such as virtual and augmented reality) where users engage with multiple perceptual channels and navigate complex environments. In this regard, Robert’s proposal [5] provides a flexible yet structured basis for evaluating digital experiences. Table 1 details each of these dimensions, which were used as the basis for proposing the evaluation methodology. Additionally, individual differences (such as a user’s familiarity with digital technologies or their level of digital self-efficacy) may influence how UX dimensions are perceived and experienced. These psychological factors can shape expectations, perceived usability, and emotional responses, especially in immersive environments that demand novel interaction skills [6,7].

### 2.2. User Experience Evaluation

Evaluating the UX of an interactive system involves measuring how well it achieves positive outcomes across these multiple dimensions. Unlike evaluations focused solely on usability (which typically measure effectiveness, efficiency, and satisfaction), UX evaluation also incorporates aspects such as emotions, level of engagement, holistic satisfaction, and other long-term effects on the user [8].

In practice, both objective and subjective methods are used to evaluate UX in different contexts [9]. On the one hand, objective metrics (such as task completion or task performance) can indicate the user’s state while interacting with the system [9]. On the other hand, subjective measures are essential to capture the user’s internal perception; these include surveys and standardized questionnaires (such as AttrakDiff [10], UEQ [11], SUS [12], among others), emotional scales, interviews, and qualitative observations dfki.de. These instruments make it possible to assess, for example, how enjoyable, intuitive, stressful, or satisfying an interactive experience is.

It is important to select the relevant UX dimensions according to the context of use. For instance, psychological dimensions (such as enjoyment or frustration) are particularly relevant in video games, whereas social dimensions may carry more weight in collaborative applications. A well-designed UX evaluation combines multiple methods to obtain a comprehensive view. In summary, UX evaluation seeks to measure the quality of the UX beyond mere usability, providing valuable information to support iterative design and ensure that the product not only functions well but also generate positive emotions and meaningful value for the user [8].

### 2.3. Immersive Environments: Virtual Reality, Augmented Reality, Mixed Reality, and Extended Reality

Virtual Reality (VR) is defined as a computer-generated simulation that immerses users in a synthetic environment by simulating their presence in a virtual space [13]. VR systems typically include head-mounted displays (HMDs), haptic devices, and auditory outputs that respond to the user’s movements and provide real-time feedback. The goal is to induce a sense of presence, allowing users to feel as though they are physically inside the virtual world. VR is extensively used in gaming, simulation, education, and industrial training [14,15].

Augmented Reality (AR) blends digital content with the physical world by overlaying virtual elements onto the user’s real environment [16]. Unlike VR, AR does not replace the real world but enhances it in real time. Key characteristics of AR include interactivity, accurate spatial registration, and seamless integration between real and virtual objects. AR is widely applied in mobile applications, education, retail, and industrial maintenance [17,18].

Mixed Reality (MR) lies on the continuum between VR and AR. It merges physical and digital environments, allowing real and virtual objects to interact in real time [19]. MR enables a two-way interaction between the user and the environment, combining spatial mapping, object anchoring, and real-world awareness. It requires more advanced hardware such as smart glasses or spatial sensors and is still in the early stages of adoption.

Extended Reality (XR) is an umbrella term encompassing VR, AR, and MR [20,21]. It describes all real-and-virtual combined environments generated by computer technology and wearables. XR emphasizes the fluidity between these modalities, allowing users to experience digital environments with varying levels of immersion and interaction. XR is increasingly relevant in education, healthcare, simulation, and collaborative platforms.

Due to the immersive capabilities of these technologies, four key characteristics are relevant for evaluating user experience in XR [13,16]:

• Immersion: The extent to which a system convincingly replicates real-world sensory input, producing a sensation of “being there”.
• Interactivity: The degree of real-time responsiveness and user influence on the environment. XR interactivity occurs in three-dimensional space and often includes gesture-based or motion-based input.
• Presence: The subjective experience of existing within a virtual space. Presence is influenced by immersion, realism, and psychological engagement.
• Imagination: Emotional and cognitive connection established by the XR environment. This includes the ability to suspend disbelief and perceive the virtual environment as real.

Understanding the features and components of XR applications was essential for developing an effective and useful UX evaluation methodology. Features such as immersion, interactivity, and presence directly influence how users perceive and engage with XR environments, while components such as input devices, output interfaces, and experience software determine the technical ways through which these interactions occur. These features guided the selection and mapping of evaluation instruments to specific UX dimensions, ensuring that the proposed methodology could accurately assess both UX and XR applications.

### 2.4. Related Work

Several previous studies have investigated UX evaluation in XR environments. In general, researchers tend to adapt traditional HCI evaluation methods to immersive contexts, combining objective and subjective metrics. For example, UX studies in VR/AR commonly collect objective measures (motion-tracking data, task completion time, or physiological indicators such as heart rate) together with subjective measures obtained through post-experience questionnaires [9]. Many standardized questionnaires originally developed for non-immersive media have been applied in XR. For instance, presence scales (Sense of Presence questionnaires [22,23]) are used to capture specific aspects of the virtual experience [9].

On the other hand, Vona et al. propose a user-centric evaluation method for XR-enhanced digital twin applications, incorporating usability, cognitive load, and engagement metrics through questionnaires and observational studies [24]. Meanwhile, Ansari introduces an automated UX assessment framework using emotion-based test agents, reducing reliance on manual testing [25]. Related to UX dimensions, Nguyen and Bednarz analyze factors affecting UX in collaborative XR, including group dynamics, avatars, nonverbal communication, and presence, identifying major research gaps in co-experience design [26]. Similarly, Izzouzi et al. highlight the importance of evaluating media-based social interactions to improve trust and engagement in XR environments [27].

However, research has pointed out that there is still no strong consensus on standardized methods for evaluating UX in these immersive environments [9]. Each study may employ different approaches, which makes it difficult to compare results and establish common best practices.

In addition, specific challenges have emerged due to the nature of XR. These include how to adequately measure factors such as presence, immersion, or “simulator” sickness, which can significantly affect the experience [28]. For example, a traditional evaluation method may require adjustments so as not to interrupt the sense of presence during a VR experiment. Some researchers have explored innovative solutions, such as integrating questionnaires directly into the virtual environment (e.g., asking questions within VR instead of removing the user from the virtual world to complete a survey) [9]. Despite these advances, a diversity of approaches remains, and unified standards for UX evaluation in XR are still lacking [9].

Several recent review studies highlight existing gaps. For instance, a systematic review on UX in Augmented Reality found that most studies employ conventional quantitative methods and that there are very few metrics or instruments designed specifically for AR [8]. In fact, this review identified only three standardized UX questionnaires specific to AR, none of which were focused on particular domains such as education or training. This suggests that practitioners often rely on generic UX surveys or adapt usability instruments, which may overlook unique aspects of the augmented experience. Similarly, in the VR domain, theoretical models have been proposed to extend traditional UX dimensions to include immersive factors. For example, Rauschnabel et al. incorporate presence and immersion as central dimensions when evaluating the acceptance of XR technologies [29].

In summary, related work highlights the need for UX evaluation methodologies adapted to XR that combine the strengths of existing approaches while covering the new dimensions involved. The methodology proposed in this article aims to address this gap by incorporating classical UX dimensions [5] and integrating them with characteristics specific to immersive environments (such as presence, immersion, interactivity, and imagination), thus achieving a more comprehensive and appropriate evaluation process for XR experiences.

## 3. Process for Developing the Methodology

**Figure 1:**
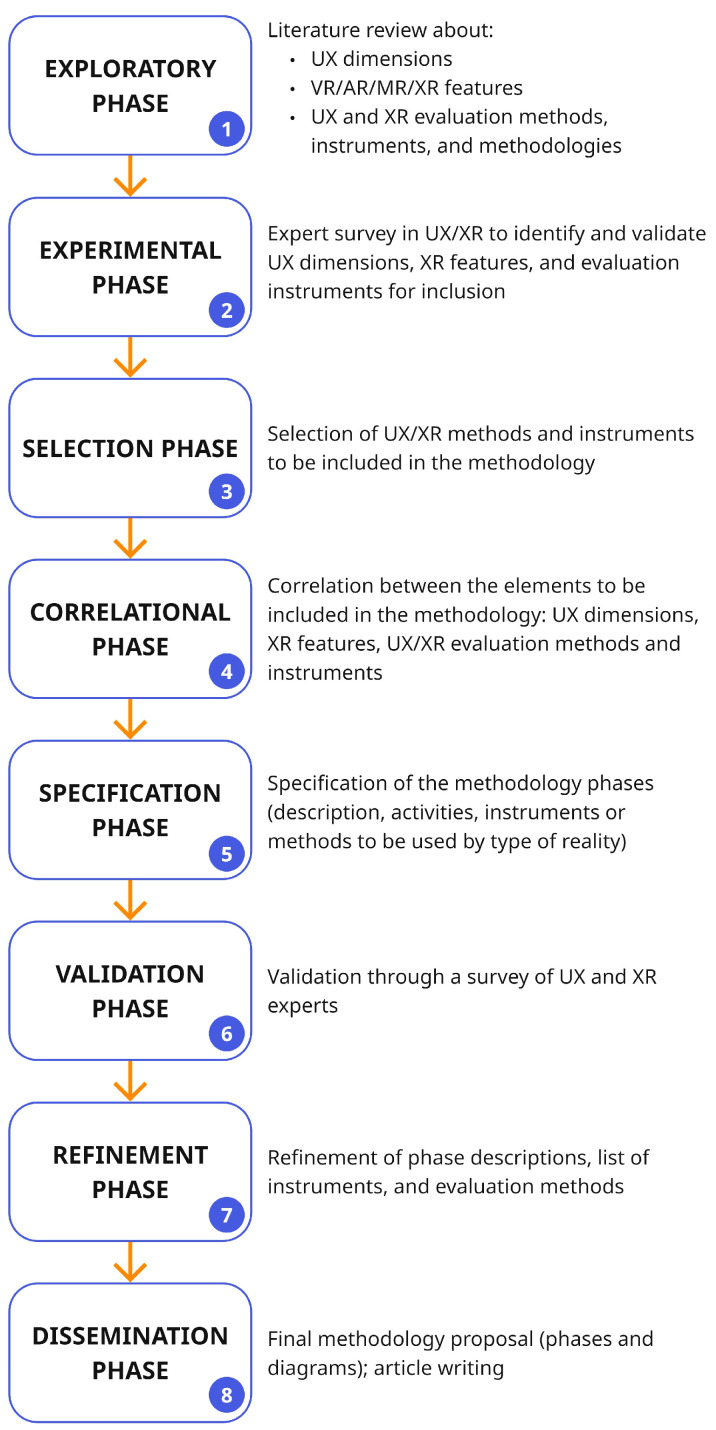
Research process for creating the methodology for evaluating user experience (UX) in Extended Reality (XR) applications.

The proposed methodology for evaluating user experience (UX) in Extended Reality (XR) applications was developed through a structured and iterative research process consisting of seven phases. The methodology applied followed the principles of Design Science Research (DSR) [30]. This paradigm supports the creation and refinement of innovative artifacts through problem identification, design, evaluation, and communication stages. The seven phases implemented in this study (exploratory, experimental, selection, correlational, specification, validation, and refinement) are aligned with the core components of DSR [30], but were adapted to fit the context of methodological construction and validation in the field of immersive technologies. Each phase contributed specific empirical or conceptual insights that shaped the final structure of the methodology. This approach ensured methodological rigor and practical applicability, combining literature review, empirical testing, expert input, and iterative refinement. To visually summarize the methodological development process, Figure 1 illustrates the seven phases followed during the creation of the UX evaluation methodology.

In the Exploratory phase, a comprehensive literature review was conducted to identify existing UX evaluation methods and instruments relevant to XR systems. The literature review followed a systematic approach based on the protocol by Kitchenham [31]. The process was carried out between 2021 and 2024 and involved three main steps: (1) planning the review (defining the research questions, databases, keywords, inclusion criteria, and data extraction strategy), (2) conducting the review (selecting articles, extracting relevant data, and synthesizing results), and (3) reporting the findings. Searches were performed in three major databases, ScienceDirect, Scopus, and the ACM Digital Library; using combinations of keywords such as “user experience”, “user experience evaluation”, “method or instrument”, “extended reality”, “virtual reality”, “augmented reality”, and “mixed reality”. The goal was to collect a wide range of standardized tools (both qualitative and quantitative) that could address traditional UX dimensions and immersive-specific characteristics. For each method, we analyzed which UX dimensions it could evaluate (based on the eight-dimension framework by Jean-Marc Robert [5]) as well as which XR characteristics it addressed: immersion, presence, interactivity, and imagination [13,16]. Two analysis tables were created: one mapping instruments to UX dimensions, and another mapping instruments to XR characteristics. This allowed us to identify a diverse set of methods that, when combined, can cover the full scope of both UX dimensions and XR-specific attributes. In addition, the methods and instruments were reviewed in terms of their theoretical basis, areas of application, and suitability for different types of XR environments (VR, AR, and MR). The results of this literature review informed the subsequent empirical evaluation with users in phase 2 (experimental Phase) and the selection of components in phase 3 (selection phase).

In the Experimental phase, a user study was conducted in a real XR environment using a “virtual bridge application” to test the practical viability of selected instruments. Participants performed interaction tasks and completed various standardized questionnaires. This experiment helped assess the cognitive load, clarity, and overall usability of the instruments and evaluation flow. Feedback from this phase informed time constraints, tool selection, and design adjustments for the methodology.

In the Selection phase, the set of candidate methods and instruments was refined. Selection criteria included empirical usability, domain relevance, and coverage of diverse UX dimensions and XR features. Instruments that demonstrated ambiguity, redundancy, or lack of relevance were excluded. This filtering process ensured that the final methodology would be both focused and flexible. In the Correlational phase, a mapping was developed linking each selected method and instrument to specific UX dimensions (as proposed by Robert) and XR characteristics (immersion, presence, interactivity, imagination). The goal was to construct a conceptual matrix that would support evaluators in selecting appropriate methods/instruments based on application type and evaluation goals. This mapping provided theoretical setting for the structure of the methodology.

In the Specification phase, the core structure of the methodology was formally defined. It includes evaluation steps, decision-making flow, and a recommended selection of instruments for pre-, during-, and post-experience stages. The methodology also incorporates subjective and objective measures, balancing quantitative validity with user-centered insight. Special care was taken to ensure clarity and flexibility, allowing adaptation to different XR contexts. In the Validation phase, a structured expert review was conducted to assess the clarity, usefulness, and applicability of the methodology. Experts in UX and HCI, with academic experience, were invited to review the methodology through a formal questionnaire. Their feedback highlighted key areas for improvement, including the definition of some dimensions and XR traits, and the relationship between evaluation phases and methods/instruments. Finally, in the Refinement phase, the methodology was refined based on expert feedback. Adjustments included clearer descriptions of XR characteristics, simplification of evaluation flows, and the integration of missing subdimensions (such as ergonomic aspects). The instrument set was revised to reduce redundancy and improve feasibility. These improvements enhanced both the robustness and the practical relevance of the final methodology.

## 4. Results

This section presents the main results obtained throughout each phase of the methodological development process. While the overall process was structured into eight iterative stages (as described in Section 3), this section focuses specifically on the empirical and analytical outcomes that directly informed the construction and refinement of the proposed UX evaluation methodology for XR applications.

The complete structure and details of the proposed UX evaluation methodology are presented separately in Section 5. This current section presents the key phases, analyses, and decisions that supported its construction and refinement.

### 4.1. Exploratory Phase

During the exploratory phase, an extensive literature review was conducted to identify UX evaluation methods and/or instruments applicable to immersive technologies. The review focused on selecting validated methods that addressed both traditional UX dimensions and XR-specific characteristics, considering the growing complexity nature of XR environments.

A total of 21 evaluation methods/instruments were identified and analyzed in terms of their relevance to: (1) the eight UX dimensions proposed by Robert [5], and (2) four key XR characteristics: immersion, presence, interactivity, and imagination.

The selection criteria included methods and instruments that had been previously applied in XR contexts or demonstrated conceptual alignment with immersive systems, even if originally designed for non-XR platforms. Table 2 summarizes the methods and instruments reviewed.

### 4.2. Experimental Phase

The experimental phase aimed to conduct preliminary user tests to evaluate the suitability, applicability, and effectiveness of selected UX evaluation instruments in XR contexts. These tests were conducted with six real users interacting with a “virtual bridge” application developed within the context of a doctoral thesis at the School of Civil Engineering, Pontificia Universidad Católica (PUCV), Chile. The “virtual bridge” application was designed as an educational tool to support the teaching of structural design concepts and civil construction principles to undergraduate civil engineering students. Its purpose was to provide an immersive learning environment where users could explore the components and behavior of bridge structures interactively. All users were civil engineering students with varying degrees of familiarity with XR technologies from PUCV.

The purpose of user testing was to validate whether the instruments selected in the “exploratory phase” could capture relevant dimensions of the UX and whether the evaluation flow was reasonable in terms of time, cognitive load, and usability.

The experimental protocol consisted of several steps. Prior to the experience, users completed a demographic questionnaire to obtain information about previous experience with XR systems and potential motion sensitivity. During the experience, participants completed a series of predefined tasks in the virtual environment. Following this, they were asked to complete three standardized questionnaires:

1. Customizable Interactions Questionnaire (CIQ) [37]: used to evaluate interaction realism (audio-related items were excluded due to lack of sound in the application).
2. NASA Raw Task Load Index (NASA-TLX) [36]: measuring perceived workload.
3. System Usability Scale (SUS) [12]: assessing general usability of the system.

In addition to the standardized instruments, four open-ended questions were included to capture qualitative feedback and highlight issues that might not be reflected in quantitative scores. The evaluation session, including task execution and questionnaires, lasted approximately 30–40 min.

The responses collected revealed useful insights about the cognitive demands of the tasks, the perceived realism of the environment, and the ease of completing interactions. Appendix A presents a detailed overview of the user testing tasks, the results obtained from each evaluation instrument, and key insights from users’ open-ended responses. The results obtained confirmed that the methodology could be implemented in realistic settings and that the selected instruments were effective in capturing multiple dimensions of UX, particularly when complemented with qualitative feedback.

Although the users who participated in this experiment were undergraduate civil engineering students and they were not UX professionals, their participation was appropriate for the objectives of this stage. Specifically, they acted as domain-informed users, capable of providing relevant insights regarding the applicability, clarity, and comprehensibility of the evaluation instruments when applied to a real XR scenario aligned with their academic background. It is important to clarify that this phase was not intended as an expert validation of the instruments, but rather as an empirical trial to explore how users from a specific context (civil engineering education) respond to different UX evaluation methods. The results of this phase were then analyzed and interpreted by the research team, composed of co-authors with expertise in user experience (UX), virtual reality (VR), and human–computer interaction. Based on this expert analysis, the most appropriate instruments were selected in phase 3. A formal expert-based validation of the entire methodology was subsequently carried out in phase 6, involving an independent group of PhD-level experts in UX and XR technologies.

### 4.3. Selection Phase

During the “selection phase”, the 21 instruments identified in the “exploratory phase” were systematically analyzed to determine their relevance, scope, and potential contribution to the proposed methodology. Each instrument was evaluated based on the UX dimensions and XR features it could assess, as well as its applicability to different types of extended reality (VR, AR, or XR in general). Rather than discarding instruments, it was decided to retain all 21 instruments, recognizing that each provides value in evaluating specific dimensions or features depending on the application context. This inclusive approach ensures that the methodology remains flexible and adaptable to a wide range of XR scenarios.

### 4.4. Correlational Phase

In the “correlational phase”, each of the 21 selected instruments was systematically mapped to the specific UX dimensions and XR characteristics it evaluates, along with the types of extended reality environments (VR, AR, or XR) where it is applicable. This mapping process resulted in a structured matrix that links every instrument with its evaluative focus, allowing for more informed and context-sensitive selection during the application of the methodology. Table 3 presents the correlation between each evaluation instrument and the UX dimensions, XR features, and types of reality (VR, AR, or XR) it is best suited to assess.

This classification was essential for the development of the methodology, as it supports evaluators in selecting appropriate instruments based on the type of application and the experiential goals of the system (e.g., increasing presence, reducing cognitive load, enhancing interactivity). Several instruments such as the UEQ, IPQ, FSS, and GEQ were found to offer multidimensional coverage, making them suitable for evaluating both usability and experiential aspects like flow, presence, and emotional engagement. Other instruments, such as the VRSQ and AARC, addressed more specific aspects such as simulator sickness or audio-based augmentation.

### 4.5. Specification Phase

In the specification phase, the structure of the UX evaluation methodology was formally defined. A multi-phase process was planned to organize the evaluation procedure in a clear and adaptable manner, aligning each stage with specific objectives, target dimensions, and appropriate instruments. For each phase of the methodology, the UX dimensions to be evaluated (based on Robert’s model [5]) and the XR features to be considered (immersion, presence, and interactivity) were identified. Furthermore, a tailored selection of instruments was assigned to each stage, categorized according to the type of XR evaluated (VR, AR, or XR in general). This structured definition enables evaluators to select appropriate instruments depending on the context and type of experience. The complete specification of the methodology (including its phases, logic, evaluation flow, and detailed instrument recommendations) is presented in Section 5.

### 4.6. Validation Phase

To validate the initial version of the proposed methodology, an expert review process was conducted using an online questionnaire delivered via Google Forms. The validation process focused on key components of the proposed methodology (see Section 5). This preliminary validation aimed to assess the clarity, usefulness, completeness, and usability of the methodology’s structure and its components, and to gather qualitative feedback for refinement.

#### 4.6.1. Validation Objective

The purpose of this preliminary validation was to assess the initial structure, clarity, and applicability of the proposed methodology through expert judgment. Although the methodology has not yet been implemented or tested in multiple real-world contexts, this initial expert review provided essential feedback for its refinement. The evaluation helped identify strengths, potential ambiguities, and areas requiring further development, ensuring that the methodology evolves on a solid foundation before broader validation and deployment.

#### 4.6.2. Design of the Instrument

A structured questionnaire was designed and divided into sections to validate the proposed methodology and its components: UX dimensions, XR characteristics, the list of evaluation instruments, the relationship between instruments and evaluation targets, and the methodological phases. Each section included Likert-scale questions (1 to 5, where 1 indicated the lowest score, and 5 indicated the highest score) for the following dimensions:

• Clarity refers to the degree to which the concepts, structure, and components of the methodology are understandable and unambiguous to the expert.
• Usefulness assesses the perceived value and relevance of each component of the methodology for its intended purpose.
• Completeness indicates whether each component of the methodology includes all the necessary elements to be considered comprehensive and sufficiently robust for practical application.
• Ease of use reflects the perceived simplicity and practicality of applying the methodology in real-world contexts, including each component and instructions.

Additionally, each section included open-ended questions to collect suggestions and qualitative feedback. The experts were asked what they would add and/or remove to each component, in addition to asking for general comments and suggestions to improve each component.

#### 4.6.3. Expert Selection

Five experts participated in the validation process. All held doctoral degrees in computer engineering and had extensive experience in UX research and evaluation. Three of the experts had specific expertise in both virtual reality and UX evaluation, while the other two specialized in UX evaluation across several interactive systems. In terms of research trajectory, three experts had approximately five years of experience in the UX field, while the remaining two had been conducting UX-related research for over a decade.

#### 4.6.4. Quantitative Results

Table 4 summarizes the quantitative results obtained from the expert evaluations. It presents the average score (on a 1–5 Likert scale) assigned to each methodological component across four dimensions: clarity, usefulness, completeness, and ease of use. These averages provide an overview of how each element of the proposed methodology was perceived in terms of conceptual soundness and practical applicability.

Experts rated the components positively in terms of usefulness, with all scores above 4.0. However, completeness received slightly lower ratings, particularly for the XR characteristics (3.4) and phase 3 (application of evaluation instruments, 3.4), indicating that further detail was needed. Ease of use scores also suggested that some components, particularly the UX dimensions (3.2) and the initial phases (phase 1, 2 and 3, with 3.8, 3.8 and 3.6 scores respectively), could benefit from improved presentation or guidance.

#### 4.6.5. Qualitative Results

Experts made several recommendations across components:

• UX Dimensions: some experts suggested adding ergonomic and disorientation-related aspects, particularly relevant to VR. They also recommended more granularity in the emotional or psychological subdimensions.
• XR Characteristics: experts asked for clearer definitions and more actionable descriptions. For instance, the “imagination” characteristic was seen as conceptually interesting but hard to evaluate in practice.
• Evaluation Instruments: while the comprehensiveness of the instrument list was praised, experts proposed indicating the evaluation context (pre-, during-, post-experience) and aligning each tool more explicitly with the corresponding phases.
• Mapping table (from phase 1): this was considered one of the most valuable components. However, experts recommended improving visual clarity and notation.
• Phases: suggestions included renaming some phases to improve intuitiveness (e.g., renaming “information gathering” to “preparing the experiments”), and clarifying the outcomes expected from each phase.

Overall, the validation phase confirmed the methodological proposal as both relevant and potentially impactful, especially due to its structured approach and multidimensional scope. The feedback highlighted opportunities to improve coherence, terminology, and evaluability, particularly in the earlier phases. These observations directly informed the refinements applied in the next phase of the methodology’s development.

### 4.7. Refinement Phase

The refinement phase involved revising and improving the proposed methodology based on the results of the expert validation. Feedback collected from both the quantitative scores and qualitative suggestions revealed specific areas where additional clarity, structure, or alignment with practical application was needed. The objective was to address these points of improvement to enhance the methodological completeness and ensure its usability across different XR evaluation contexts. Table 5 summarizes the key areas for improvement identified during the expert review, along with the refinement actions implemented for each methodological component. The final version of these components is detailed in Section 5.

## 5. Methodology for Evaluating UX in Extended Reality

**Figure 2:**
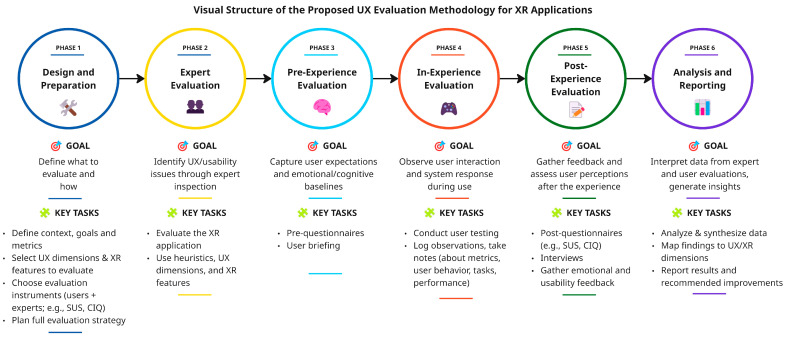
Visual structure of the proposed methodology for evaluating UX in human-centered XR applications.

This section presents the methodology for evaluating user experience (UX) in Extended Reality (XR) applications. The methodology is structured into six sequential phases that follow the natural flow of an XR user experience: preparation, expert evaluation, user evaluation (pre-experience, during-experience, post-experience), and result interpretation. It is designed to be modular and adaptable across different types of XR (VR, AR, MR) and user-centered application domains. Figure 2 illustrates the six-phase structure of the methodology, providing an overview of its goals and key tasks across the XR experience cycle. Each phase is described below in terms of its objectives, scope, key activities, inputs and outputs, and applicable instruments when relevant.

### 5.1. Phase 1: Design and Preparation

• **Objective:** To define the scope and context of the evaluation, design the immersive experience, and select the appropriate UX dimensions, XR characteristics, and evaluation instruments according to the modality (VR, AR, MR) and experience goals.
• **Description:** This phase establishes the foundation for the entire evaluation process. It involves defining the context of the XR experience to be evaluated, identifying key UX dimensions and XR characteristics to be measured, selecting appropriate instruments, and designing the evaluation protocols to be applied with users and experts. This includes preparing both the technical setup and the evaluation tools tailored to the specific XR modality (VR, AR, or MR).
• **Main Activities:**
  ○ Define evaluation goals, context of use, and target user profile.
  ○ Select relevant UX dimensions (e.g., usability, cognitive, emotional) and XR features (e.g., immersion, presence).
  ○ Choose evaluation instruments appropriate for the target modality (VR, AR, or XR).
  ○ Design user evaluation flow: pre-, in-, and post-experience assessments.
  ○ Plan expert evaluation activities (to be applied in Phase 2).
  ○ Prepare technical setup and materials for data collection.
• **Inputs:**
  ○ XR application (functional application, prototype or concept).
  ○ User characteristics (for selecting representative users).
  ○ Evaluation objectives.
  ○ Instrument repository.
• **Outputs:**
  ○ Evaluation plan document (including goals, UX dimensions, XR features, and selected instruments).
  ○ Set of instruments prepared for each phase of user evaluation.
  ○ Materials and protocols for expert evaluation (used in Phase 2).
• **Applicable Instruments:** Instruments are selected according to the type of XR to be evaluated. Table 6 summarizes the tools categorized by modality. A detailed overview of the 21 evaluation instruments, including their characteristics, application methods, and applicability across XR modalities, is provided in Appendix B.

### 5.2. Phase 2: Expert Evaluation

• **Objective:** To identify usability and UX problems in the XR application through structured inspection by domain experts, based on established evaluation methods or frameworks and guided by predefined UX dimensions and XR attributes.
• **Description:** This phase involves a formal evaluation of XR experience by a group of expert reviewers. The goal is to uncover design or usability/UX problems prior to testing with users, ensuring that the application aligns with best practices in interaction, clarity, and overall experience. The evaluation does not focus on refining the experimental design but rather on assessing the application itself. Experts may use heuristic methods, structured walkthroughs, or surveys, guided by the UX dimensions and XR features identified in Phase 1.
• **Main Activities:**
  ○ Recruit domain experts with experience in UX and/or XR.
  ○ Provide experts with evaluation guidelines based on selected UX dimensions and XR attributes.
  ○ Apply expert-based methods (e.g., heuristic evaluation, formal inspection, structured survey).
  ○ Document expert findings, both quantitatively and qualitatively.
  ○ Consolidate insights into actionable feedback for potential improvements.
• **Inputs:**
  ○ Selected evaluation UX dimensions and XR features from Phase 1;
  ○ XR prototype or functional application;
  ○ Expert selection criteria and recruitment list;
  ○ Instruments or checklists for expert evaluation.
• **Outputs:**
  ○ Structured feedback and reports from experts;
  ○ Identified usability and UX problems;
  ○ Suggested improvements to refine the XR experience;
  ○ Adjustments to the evaluation plan (if needed).

### 5.3. Phase 3: Pre-Experience Evaluation

• **Objective:** To capture the user’s initial perceptions, expectations, and emotional or cognitive states before engaging with the XR experience.
• **Description:** This phase focuses on gathering contextual and baseline information from users prior to their interaction with the XR environment. It aims to evaluate their expectations, readiness, and prior experiences, which can affect how they perceive and interact with the immersive system. A useful component of this phase is collecting demographic and background data (such as age, gender, educational background, and prior experience with XR technologies, or the specific application domain). This contextual information is essential for interpreting later findings and ensuring that the sample characteristics are well documented.
• **Main Activities:**
  ○ Present the evaluation purpose and obtain informed consent.
  ○ Collect demographic and background information relevant to the study (e.g., age, gender, prior XR exposure, familiarity with the task domain).
  ○ Measure baseline states (e.g., stress, attention, motivation).
  ○ Gather user expectations about usability, usefulness, immersion, among others.
  ○ Apply pre-experience questionnaires or brief interviews.
• **Inputs:**
  ○ Final version of the evaluation protocol (from Phase 1);
  ○ User recruitment criteria and consent forms;
  ○ Pre-experience instruments selected;
  ○ Baseline and demographic data forms.
• **Outputs:**
  ○ Pre-experience user data (quantitative and qualitative);
  ○ Baseline measures of user state;
  ○ Demographic and contextual user profiles;
  ○ Insights into user expectations and preconceptions.

### 5.4. Phase 4: In-Experience Evaluation

• **Objective:** To collect data on the user’s real-time interaction, behavior, and perception during the XR experience, capturing experiential and performance-related aspects.
• **Description:** This phase focuses on monitoring the user as they interact with the XR application. The goal is to understand how users navigate, perform tasks, and emotionally respond within the immersive environment. Both objective and subjective data can be gathered, including task performance metrics, system logs, real-time feedback, and observational notes. Some instruments can be embedded within the XR experience to minimize disruption, while others rely on passive monitoring or brief prompts. The evaluation may involve either passive observation or guided facilitation. In guided sessions, a moderator can intervene with neutral, non-leading questions to help users progress through tasks or to probe deeper into specific reactions. Alternatively, autonomous sessions may be used, where users are given a usage scenario and a set of tasks to complete independently.
• **Main Activities:**
  ○ Monitor user interaction with the XR environment (live or recorded).
  ○ Track task performance and collect system usage metrics (e.g., task completion time, errors, navigation paths).
  ○ Apply embedded or real-time instruments (e.g., flow prompts, interaction logs).
  ○ Observe user behavior, reactions, and gestures qualitatively.
  ○ Choose an interaction mode:
    ▪ Moderated: The facilitator can ask neutral guiding questions to clarify user actions or collect qualitative impressions during task execution.
    ▪ Unmoderated: The user completes a predefined usage scenario and task set independently, using written or voice instructions.
• **Inputs:**
  ○ XR experience prototype with finalized tasks and scenario;
  ○ Selected in-experience instruments and data collection tools;
  ○ Observation protocols or screen/audio recording tools;
  ○ Scenario description and task instructions.
• **Outputs:**
  ○ Performance and interaction data/metrics;
  ○ Qualitative observations and user responses;
  ○ Real-time user reactions and feedback;
  ○ Evidence of immersion, presence, interactivity, and usability.

### 5.5. Phase 5: Post-Experience Evaluation

• **Objective:** To assess the user’s perception of the XR experience after interaction, capturing usability, satisfaction, emotional responses, perceived workload, presence, and overall quality of the experience.
• **Description:** This phase focuses on gathering reflective feedback from users after they have completed the XR experience. It complements the data collected during interaction by providing insights into subjective impressions, emotional impact, perceived usability, and engagement. A combination of standardized questionnaires, open-ended questions, and post-session interviews can be used. The choice of instruments depends on the specific dimensions of UX and XR features previously defined.
• **Main Activities:**
  ○ Apply post-experience questionnaires (e.g., SUS, UEQ, AttrakDiff, PQ, IPQ, GEQ, IEQ, etc.).
  ○ Conduct short interviews to gather qualitative impressions and user suggestions.
  ○ Collect user ratings on specific UX dimensions and XR attributes.
  ○ Review symptoms of discomfort or fatigue (e.g., cybersickness, mental load).
  ○ Optional: ask users to reflect on their expectations vs. experience.
• **Inputs:**
  ○ Completed interaction session (from Phase 4);
  ○ Selected post-experience instruments;
  ○ Questionnaire administration tools (paper, digital);
  ○ Interview protocol.
• **Outputs:**
  ○ Subjective evaluation data (quantitative and qualitative);
  ○ User feedback on usability, enjoyment, and immersion;
  ○ Perceptions of presence, interactivity, and imagination;
  ○ Suggestions for system improvement.

### 5.6. Phase 6: Analysis and Reporting

• **Objective:** To consolidate, interpret, and report the results obtained from expert and user evaluations in order to extract actionable insights and support decision-making in the design and improvement of XR applications.
• **Description:** This final phase focuses on organizing and analyzing all data collected throughout the evaluation process (from expert feedback, pre-experience data, in-experience metrics, to post-experience perceptions). It includes both quantitative (e.g., scores, performance indicators) and qualitative (e.g., interview responses, observations) analysis. The goal is to derive conclusions about UX and XR application quality, identify strengths and weaknesses, and generate improvement recommendations. Results are synthesized into a final report or presentation, which may also be used to inform subsequent development cycles or academic dissemination.
• **Main Activities:**
  ○ Organize data from all evaluation phases (experts and users).
  ○ Perform statistical and thematic analysis depending on data type (e.g., scores from usability questionnaires, task completion times, presence or immersion ratings, open-ended feedback, emotional reactions, demographic differences such as gender or prior XR experience).
  ○ Compare results across instruments and dimensions (UX and XR attributes).
  ○ Visualize results in charts, tables, and summaries.
  ○ Draft a comprehensive evaluation report or presentation.
  ○ Formulate actionable recommendations for improvement.
• **Inputs:**
  ○ Evaluation data from Phases 2 to 5;
  ○ Defined UX dimensions and XR attributes (from Phase 1);
  ○ Notes and insights from facilitators or evaluators;
  ○ Analysis framework (e.g., statistical tools, coding guides).
• **Outputs:**
  ○ Consolidated findings and interpretation;
  ○ Evaluation report with evidence and insights;
  ○ Visual representations of key results;
  ○ Design recommendations and future directions.

## 6. Discussions

### 6.1. Interpretation of Results

The development and validation process of the proposed methodology highlighted the multidimensional nature of user experience (UX) in immersive environments. The eight UX dimensions adopted from Jean-Marc Robert, along with the four XR-specific attributes (immersion, presence, interactivity, and imagination), proved to be comprehensive and complementary in capturing the core elements of user interaction within extended reality (XR) applications. The selection of 21 instruments (grouped by XR modality) demonstrated that no single method suffices to address the full spectrum of experiential, cognitive, and sensory aspects involved. The expert evaluation confirmed the methodological clarity, usefulness, and completeness, reinforcing the need for a structured, phase-based approach to ensure rigorous and replicable assessments.

Furthermore, the refined version of the methodology (with six sequential phases) reflects a clear alignment between design preparation, expert validation, user-centered evaluation, and the consolidation of results. This alignment is critical for ensuring that assessments are not only reliable but also actionable for designers, developers, and researchers working in XR environments. In line with the biomimetic perspective, the methodology also draws inspiration from the way living organisms perceive, adapt, and interact with complex environments, guiding the evaluation of XR systems as adaptive, multisensory experiences that respond dynamically to human perception and behavior.

### 6.2. Comparison with Previous Studies

Recent literature reviews on UX in VR, AR, and XR contexts have consistently pointed to the lack of unified frameworks for experience evaluation. Many prior studies rely on adaptations of traditional HCI instruments, such as SUS or NASA-TLX, without fully addressing the unique characteristics of immersive environments. While some researchers propose hybrid methods or include presence scales (e.g., PQ, IPQ), most studies lack a structured process that guides evaluation across the temporal stages of the user journey. Compared to these approaches, the methodology proposed here offers an integrated, modular, and scalable process that considers both subjective and objective dimensions. By including expert evaluation as an independent phase, the methodology introduces a proactive filter that identifies usability issues before user testing, something not commonly found in prior models. Additionally, the explicit use of presence, immersion, and interactivity as dimensions is novel and aligns with XR’s creative and experiential potential, often overlooked in usability-focused evaluations.

### 6.3. Toward Real-World Validation

While this study focuses on the development and expert validation of the proposed UX evaluation methodology, we acknowledge the importance of demonstrating its applicability in real-world XR scenarios. As an initial and preliminary example, the methodology was partially applied in a pilot study using the “Virtual Bridge” application—a VR tool designed to support civil engineering students in learning about structural design. This use case served as an early testing ground to explore how UX and XR attributes can be systematically evaluated within an educational setting. Although limited in scope, the study highlighted the practical utility of the methodology and informed some of its refinements.

Looking forward, we plan to implement a full-scale validation of the methodology in a real educational context using a new VR application for teaching geometry and mathematics to middle and high school students. This planned study will involve diverse user profiles, including varying levels of digital experience and educational backgrounds. Data will be collected through multiple instruments across all phases of the methodology, enabling a comprehensive evaluation of both the usability and its ability to capture rich user experience insights in immersive learning environments. This future step will be key to assessing the method’s robustness, adaptability, and impact in applied settings.

### 6.4. Biomimetic Interpretations of Interactivity in XR

A key attribute of immersive XR experiences is interactivity, which allows users to act upon the virtual environment and perceive meaningful changes in response. From a biomimetic perspective, this mirrors the way biological organisms engage in dynamic exchanges with their environment through affordances (opportunities for action perceived through sensory input) and homeostatic feedback mechanisms, which enable adaptive responses that maintain internal equilibrium. When XR systems respond smoothly to user actions and allow for real-time environmental adaptation, they emulate these biological feedback loops, reinforcing a sense of naturalness and intuitive engagement. Acknowledging this parallel provides deeper scientific grounding for the methodological emphasis on interactivity as a core evaluative dimension.

### 6.5. Practical Implications

This methodology provides a replicable structure that can be applied across diverse XR domains, including education, healthcare, industrial training, and cultural heritage. Its flexibility allows practitioners to adapt the instrument set according to the type of reality (VR, AR, MR) and the specific use case, while still maintaining methodological consistency. Designers can benefit from the phase-based approach to plan evaluations at different stages of the product lifecycle, before deployment, during beta testing, and after user exposure. Moreover, the inclusion of an expert-focused phase helps reduce design flaws early in the process, potentially saving time and resources in later stages. The tables, taxonomy of instruments, and detailed description of each phase provide a practical toolkit for UX researchers and developers working in immersive technologies.

### 6.6. Limitations

Despite its comprehensiveness, the current version of the methodology has some limitations. First, its validation has been preliminary and limited to expert review. While expert feedback was highly favorable, the methodology has yet to be applied and tested in large-scale, real-world XR projects across different domains. Second, the quantitative expert evaluation (e.g., clarity, usefulness, completeness, and ease of use) was conducted only once (prior to the refinement phase). While the refinement stage incorporated expert feedback and addressed low-scoring components, time constraints prevented a follow-up quantitative assessment using the same scale. As a result, the validation presented in this study should be considered preliminary. Future work should include a second round of quantitative evaluation to assess whether the refinements have led to measurable improvements. On other hand, while the methodology is structured to guide evaluations, it does not replace the need for thoughtful adaptation to specific use cases, including cultural, demographic, or technological constraints. In addition, the number of instruments and phases may require significant time and planning resources. For small teams or fast-paced development cycles, applying the full methodology may be challenging unless tools are developed to automate or support its implementation.

## 7. Conclusions and Future Work

This article presents a comprehensive and structured methodology for evaluating user experience in human-centered extended reality (XR) applications. The proposed approach integrates classical UX dimensions with XR-specific attributes and is organized into six sequential phases: Design and Preparation, Expert Evaluation, Pre-Experience Evaluation, In-Experience Evaluation, Post-Experience Evaluation, and Analysis and Reporting. This structure ensures a holistic, multidimensional, and replicable evaluation process that supports both formative and summative assessments.

The methodology was developed through an iterative process that included extensive literature review, expert validation, and refinement. It offers clear guidance on the selection of evaluation instruments, tailored to different XR modalities (VR, AR, MR), and provides practical tools to support implementation. The expert validation phase demonstrated the methodological clarity, usefulness, and completeness of the framework, reinforcing its potential for adoption in both research and applied contexts. Additionally, the methodology aligns with biomimetic principles by treating immersive systems as experiential ecosystems, where user interaction mirrors natural processes of perception, feedback, and adaptation, opening avenues for more intuitive and human-centered XR design.

However, the current version represents an initial validated proposal. As such, further empirical validation is needed through application in real XR projects, with diverse user groups and across different domains (e.g., education, healthcare, industry). These implementations would help assess its generalizability, usability in practice, and adaptability to various development settings. Future research should focus on the following directions:

• Empirical validation in real-world contexts, applying the methodology with actual users and use cases to test its effectiveness and scalability.
• Development of digital tools or platforms that assist in applying the methodology, automating data collection, instrument selection, and results reporting (e.g., a digital dashboard that automates the selection of UX evaluation instruments that guide practitioners based on inputs such as XR modality, evaluation phase, and desired UX dimensions).
• Customization for specific domains, such as training simulators, medical XR, or gamified learning environments, where domain-specific metrics may enhance evaluation depth.
• Incorporation of emerging dimensions, such as ethical considerations, privacy perceptions, accessibility, and sustainability in XR environments.
• Longitudinal UX evaluation, exploring how user experience evolves over time in repeated or prolonged XR usage.
• Exploration of individual traits and psychological readiness, such as digital self-efficacy, to better understand how personal factors influence the user’s ability to engage with and benefit from XR experiences. Integrating such variables could strengthen the human-centered adaptability of the methodology and support more inclusive evaluation strategies.
• Explore alternative constructs for measuring imagination. Given the abstract nature of the imagination attribute, future research could examine its alignment with validated constructs such as “cognitive absorption” or “narrative engagement”. This may support more standardized assessment of deep user engagement in XR environments, especially those with narrative or exploratory components.
• Examine how the methodology performs in diverse cultural and regional contexts (especially in underrepresented areas) where norms, digital literacy, and interaction patterns may influence UX in XR environments.

By addressing these future directions, the methodology can be further strengthened and expanded into a robust standard for immersive UX evaluation in both academic and industry settings.

## Figures and Tables

**Table 1:** User experience dimensions proposed by Jean-Marc Robert [5].

Dimension	Description
Functional	“Qualities that make a product reliable, compatible with others, accessible, available, and well adapted to its physical and human environment” [5].
Usefulness/Usability	“It includes usefulness (quality of a product that enables the user to satisfy his/her needs and achieve his/her objectives), usability (quality of a product that is easy to learn and use), and performance characteristics (that includes response speed, memory capacity, computing power, and image quality)” [5].
Informational	“Utility, right balance, and appropriateness of the information pro-vided by the product depending on the context” [5]. It includes two sub-dimensions: quality of information and quantity of information.
Physical characteristics	“They include, for example, weight, shape, the dimensions (e.g., keyboard, display), and battery life” [5].
Sensorial/Perceptual	“Impression left by the product on the sense organs, to the impact on the user’s perception” [5]. It includes three subdimensions: visual, hearing, and tactile.
Cognitive	“Human information processing done while using the product; it includes different types of activities such as analyzing, evaluating, reflecting, learning, and creating” [5].
Psychological	“Emotions felt by the user when s/he interacts with the product, and to the values and opinions that this interaction triggers” [5]. It includes several sub-dimensions: stress, pride, pleasure, frustration, evocation, attachment, and moral value.
Social	“Linking the user with other people through the product” [5]. It includes two sub-dimensions: contact and culture.

**Table 2:** UX evaluation methods or instruments reviewed applicable to immersive technologies.

Nº	Method/Instrument
1	Thinking Aloud [32]
2	Heuristic evaluation [33]
3	Expert evaluation [34]
4	Task Completion Time (TCT) [35]
5	AttrakDiff [10]
6	System Usability Scale (SUS) [12]
7	User Experience Questionnaire (UEQ) [11]
8	NASA-TLX [36]
9	Customizable Interactions Questionnaire (CIQ) [37]
10	“Interaction Realism” Questionnaire [38]
11	Igroup Presence Questionnaire (IPQ) [39]
12	Virtual Reality Sickness Questionnaire (VRSQ) [40]
13	Audio Augmented Reality Checklist (AARC) [41]
14	Flow Short Scale (FSS) [42]
15	ITC—Sense of Presence Inventory [22]
16	Game Experience Questionnaire (GEQ) [43]
17	Presence Questionnaire (PQ) [23]
18	After Scenario Questionnaire (ASQ) [44]
19	Post-Study System Usability Questionnaire (PSSUQ) [45]
20	User Engagement Scale (UES) [46]
21	Immersion Experience Questionnaire (IEQ) [47]

**Table 3:** Mapping of UX evaluation instruments to UX dimensions, XR features, and applicable types of reality.

Nº	Instrument	UX Dimensions Evaluated	XR Features Covered	Applicable Reality Type
1	Thinking Aloud [32]	Usability, Informational, Cognitive, Sensorial/Perceptual	Immersion, Interactivity, Presence	XR
2	Heuristic evaluation [33]	Usability, Informational, Cognitive, Sensorial/Perceptual	Immersion, Interactivity, Presence	XR
3	Expert evaluation [34]	Usability, Informational, Cognitive, Sensorial/Perceptual	Immersion, Interactivity, Presence	XR
4	Task Completion Time (TCT) [35]	Usability	Interactivity	XR
5	AttrakDiff [10]	Usability	Interactivity	XR
6	System Usability Scale (SUS) [12]	Usability	Interactivity	VR, AR
7	User Experience Questionnaire (UEQ) [11]	Functional, Physical, Sensorial/Perceptual, Psychological	Immersion, Interactivity, Presence	VR, AR
8	NASA-TLX [36]	Informational, Cognitive, Psychological	Immersion, Interactivity	XR
9	Customizable Interactions Questionnaire (CIQ) [37]	Functional, Physical, Sensorial/Perceptual, Psychological	Immersion, Interactivity, Presence	VR
10	“Interaction Realism” Questionnaire [38]	Sensorial/Perceptual, Physical	Immersion, Interactivity, Presence	MR
11	Igroup Presence Questionnaire (IPQ) [39]	Sensorial/Perceptual	Immersion, Interactivity, Presence	VR, MR
12	Virtual Reality Sickness Questionnaire (VRSQ) [40]	Sensorial/Perceptual	Immersion, Interactivity, Presence	VR
13	Audio Augmented Reality Checklist (AARC) [41]	Sensorial/Perceptual	Immersion, Presence	AR
14	Flow Short Scale (FSS) [42]	Informational, Cognitive, Psychological	Immersion, Interactivity	XR
15	ITC—Sense of Presence Inventory [22]	Sensorial/Perceptual, Psychological	Immersion, Interactivity, Presence	XR
16	Game Experience Questionnaire (GEQ) [43]	Informational, Cognitive, Sensorial/Perceptual, Psychological	Immersion, Interactivity, Presence	VR
17	Presence Questionnaire (PQ) [23]	Informational, Cognitive, Psychological	Immersion, Interactivity	XR
18	After Scenario Questionnaire (ASQ) [44]	Usability, Informational, Cognitive	Interactivity	XR
19	Post-Study System Usability Questionnaire (PSSUQ) [45]	Usability, Informational, Cognitive	Immersion, Interactivity	XR
20	User Engagement Scale (UES) [46]	Functional, Physical, Psychological	Immersion	XR
21	Immersion Experience Questionnaire (IEQ) [47]	Psychological	Immersion	VR

**Table 4:** Expert evaluation scores for each methodological component across four dimensions.

Component	Clarity	Usefulness	Completeness	Ease of Use
UX dimensions	4.0	5.0	3.8	3.2
XR characteristics	4.4	4.8	3.4	3.8
Evaluation instruments list	3.6	4.8	3.6	4.0
Mapping instruments–dimensions–characteristics	4.2	4.6	4.4	4.4
Methodological phase 1 (information gathering)	3.8	4.8	3.4	3.8
Methodological phase 2 (conducting experiments)	4.2	4.8	3.8	3.8
Methodological phase 3 (application of evaluation instruments)	4.0	4.8	3.6	3.6
Methodological phase 4 (results analysis)	3.8	4.0	2.8	4.0

**Table 5:** Refinement actions for each component of the methodology.

Component	Identified Improvement Area	Refinement Action
UX dimensions	Inclusion of ergonomic and disorientation aspects; need for more emotional/psychological granularity.	Expanded psychological and sensory dimensions.
XR characteristics	Lack of actionable definitions and concrete examples; “imagination” dimension was seen as ambiguous.	Reworded definitions with clearer, measurable phrasing and included practical usage examples.
Evaluation instruments	Excessive number of instruments; unclear application context (e.g., when and how to apply).	Grouped instruments by evaluation phase and XR type; added usage recommendations for each instrument.
Instrument–Dimension–Characteristic mapping	Visual complexity and lack of explicit guidance for interpretation.	Improved visual layout and added column headers and legends for clarity.
Methodological phases	Ambiguous naming and insufficient clarity on phase deliverables and sequence.	Renamed phases for clarity; added detailed description of steps, expected outputs, and integration logic; separate activities in more phases for better understanding.

**Table 6:** UX and XR evaluation instruments included in the methodology.

Modality	Instruments
Virtual reality	IPQ, VRSQ, GEQ, IEQ, CIQ
Augmented reality	AARC
Mixed reality	Interaction Realism Questionnaire, IPQ
Any XR reality (general)	Thinking Aloud, Heuristic Evaluation, Expert Evaluation, AttrakDiff, ASQ, PSSUQ, UES, Task Completion Time (TCT), SUS, UEQ, NASA-TLX, FSS, ITC, PQ

## Data Availability

The original contributions presented in the study are included in the article, further inquiries can be directed to the corresponding author.

## Appendix A

**Table A1:** Summary of results from the “experimental phase”.

Task	Instrument	Results	Summary of Open-Ended Responses
1. Explore structural components of bridge	NASA-TLX	Medium overall workload. High on mental demand; low physical and temporal demand.	Users found the activity intellectually engaging but not overwhelming.
	SUS	Average score: 72.5 (out of 100), “Good” usability.	Interface was mostly intuitive, but some icons lacked clarity.
	CIQ	High perceived realism in object manipulation.	Users noted the visuals felt realistic, though absence of sound reduced immersion.
2. Identify load-bearing elements	NASA-TLX	Increased mental demand and effort reported; moderate frustration.	Some users found the task challenging due to unfamiliar structural terminology.
	SUS	Slight drop in usability (avg. 68.3), mainly due to unclear instructions.	Suggested adding contextual hints or labels.
	CIQ	Realism still rated high, but with lower confidence in task precision.	Users requested better feedback for task completion confirmation.
3. Simulate stress on key structures	NASA-TLX	High performance satisfaction; moderate effort and frustration.	Users enjoyed experimenting, but some found the sliders confusing.
	SUS	Average score: 70.1, consistent usability.	The interface responded well, though some lag was reported.
	CIQ	Realism perceived as strong; clear correlation between action and response.	Users appreciated being able to “see” the impact of their actions immediately.

## Appendix B

**Table A2:** Input for Phase 1 (Design and Preparation): UX/XR Evaluation Instruments.

Instrument Name	Description	Application Method	Estimated Duration	Suggested Participants	UX Dimension Evaluated	XR Attribute Evaluated	Applicable XR Modality
Thinking Aloud [32]	Participants verbalize thoughts while interacting	Real-time verbal protocol during tasks	15–30 min (typical)	5–8 users	Usability, Informational, Cognitive, Sensorial/Perceptual	Immersion, Interactivity, Presence	XR
Heuristic evaluation [33]	Experts evaluate UI using established heuristics	Review against Nielsen or XR-specific heuristics	1–3 h per expert	3–5 experts	Usability, Informational, Cognitive, Sensorial/Perceptual	Immersion, Interactivity, Presence	XR
Expert evaluation [34]	Experts evaluate based on experience	Formal inspection or structured survey	1–2 h per expert	3–5 experts	Usability, Informational, Cognitive, Sensorial/Perceptual	Immersion, Interactivity, Presence	XR
Task Completion Time (TCT) [35]	Measures how long users take to complete tasks	Automated or manual time logging	Varies by task	5–8 users	Usability	Interactivity	XR
AttrakDiff [10]	Assesses pragmatic and hedonic UX aspects	28-item questionnaire	10–15 min	10–15 users	Usability	Interactivity	XR
System Usability Scale (SUS) [12]	Measures perceived usability	10-item questionnaire	5–10 min	10–15 users	Usability	Interactivity	VR, AR
User Experience Questionnaire (UEQ) [11]	Evaluates 6 dimensions related to user experience	26-item questionnaire	10–15 min	10–15 users	Functional, Physical, Sensorial/Perceptual, Psychological	Immersion, Interactivity, Presence	VR, AR
NASA-TLX [36]	Evaluates perceived workload	6-subscale questionnaire	5–10 min	10–15 users	Informational, Cognitive, Psychological	Immersion, Interactivity	XR
Customizable Interactions Questionnaire (CIQ) [37]	Evaluates interaction quality	17-item questionnaire	5–10 min	5–10 users	Functional, Physical, Sensorial/Perceptual, Psychological	Immersion, Interactivity, Presence	VR
“Interaction Realism” Questionnaire [38]	Assesses realism in virtual interactions	7-item questionnaire	5–10 min	5–10 users	Sensorial/Perceptual, Physical	Immersion, Interactivity, Presence	MR
Igroup Presence Questionnaire (IPQ) [39]	Measures spatial presence in VR	14-item questionnaire	5–10 min	5–10 users	Sensorial/Perceptual	Immersion, Interactivity, Presence	VR, MR
Virtual Reality Sickness Questionnaire (VRSQ) [40]	Assesses simulator sickness symptoms	9-item questionnaire	5–10 min	5–10 users	Sensorial/Perceptual	Immersion, Interactivity, Presence	VR
Audio Augmented Reality Checklist (AARC) [41]	Evaluates auditory aspects in AR	9 items/heuristics	5–10 min	3–5 users	Sensorial/Perceptual	Immersion, Presence	AR
Flow Short Scale (FSS) [42]	Measures flow state during the experience	16-item scale	5–10 min	5–10 users	Informational, Cognitive, Psychological	Immersion, Interactivity	XR
ITC—Sense of Presence Inventory [22]	Measures media presence perception	44-item questionnaire	10–15 min	5–10 users	Sensorial/Perceptual, Psychological	Immersion, Interactivity, Presence	XR
Game Experience Questionnaire (GEQ) [43]	Assesses game-specific UX	33-item questionnaire	10–15 min	8–10 users	Informational, Cognitive, Sensorial/Perceptual, Psychological	Immersion, Interactivity, Presence	VR
Presence Questionnaire (PQ) [23]	Measures user’s sense of “being there”	32-item questionnaire	10–15 min	5–10 users	Informational, Cognitive, Psychological	Immersion, Interactivity	XR
After Scenario Questionnaire (ASQ) [44]	Evaluates task-specific satisfaction	3-item scale	2–5 min	10–15 users	Usability, Informational, Cognitive	Interactivity	XR
Post-Study System Usability Questionnaire (PSSUQ) [45]	Measures overall system usability	19-item questionnaire	5–10 min	10–15 users	Usability, Informational, Cognitive	Immersion, Interactivity	XR
User Engagement Scale (UES) [46]	Assesses user engagement and involvement	31-item scale	10–15 min	8–12 users	Functional, Physical, Psychological	Immersion	XR
Immersion Experience Questionnaire (IEQ) [47]	Measures immersion in interactive media	33-item questionnaire	10–15 min	8–12 users	Psychological	Immersion	VR
